# Supplementary material for: Breeding Value Estimation Based on Morphological Evaluation of the Maremmano Horse Population through Factor Analysis
Source: Animals (Basel). 2024 Jul 31;14(15):2232. doi: 10.3390/ani14152232 (PMC11310958; doi:10.3390/ani14152232)
Supplement: Supplementary file 1 [file animals-14-02232-s001.zip › Additional_Figure_1.pdf]

Figure S1: National Association of Maremmano Breeders' official continuous evaluation scale recording form.

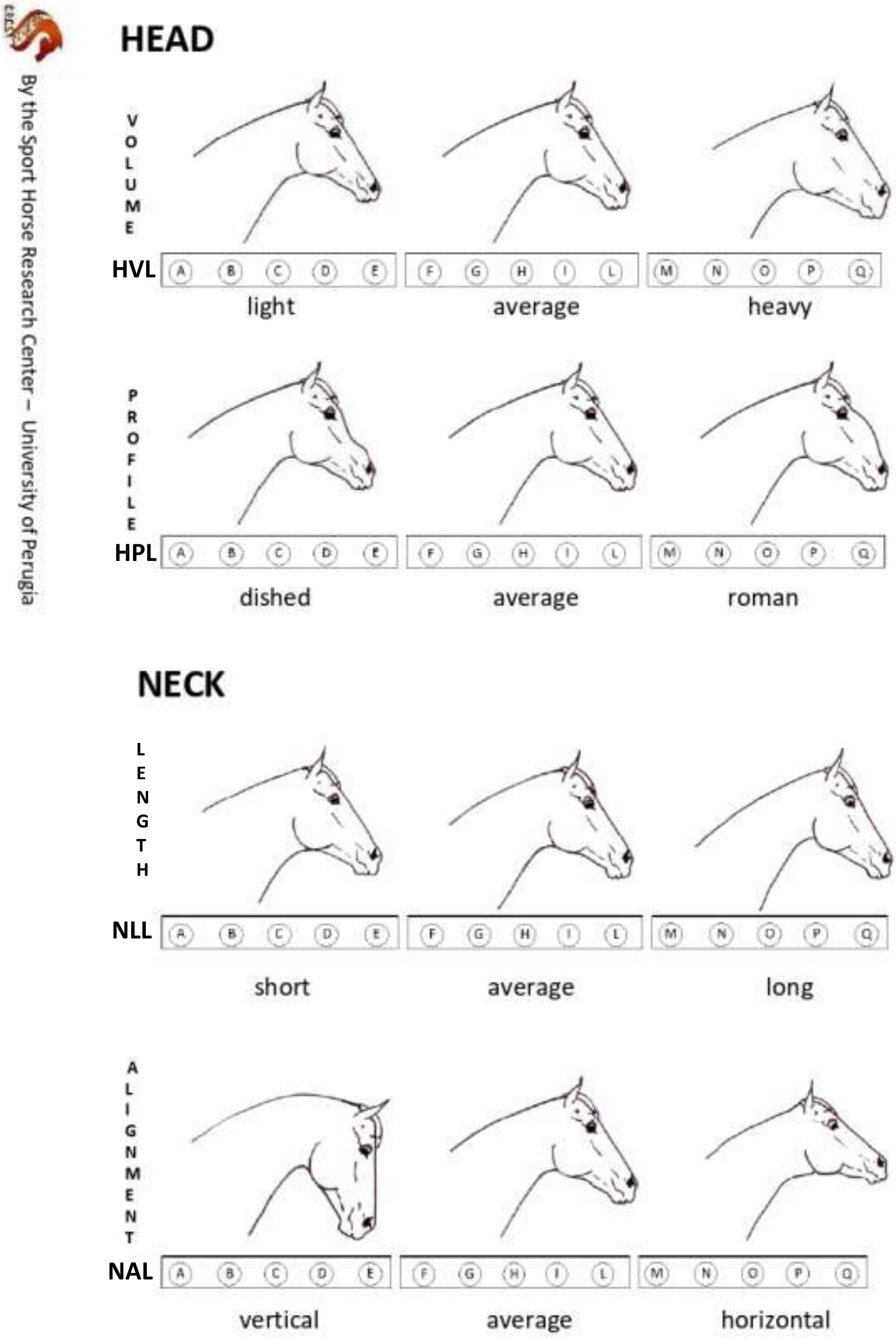

By the Sport Horse Research Center – University of Perugia

SHOULDER

LENGTH

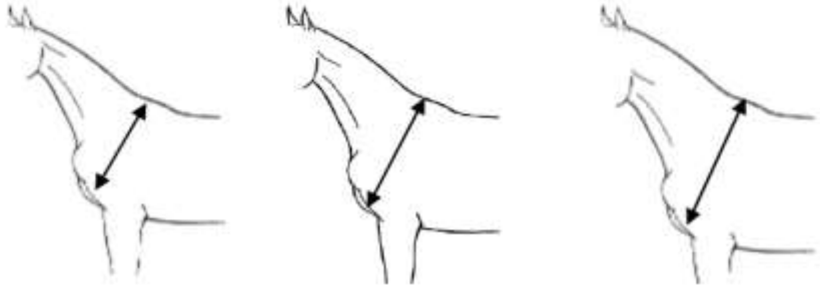

SLL	(A) (B) (C) (D) (E)	(F) (G) (H) (I) (L)	(M) (N) (O) (P) (Q)
	short	average	long

POSITION

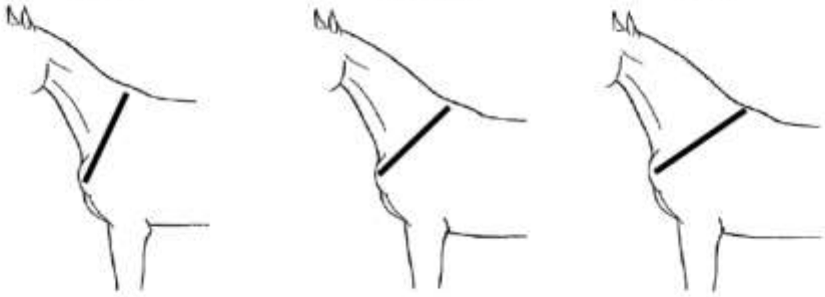

SPLC	(A) (B) (C) (D) (E)	(F) (G) (H) (I) (L)	(M) (N) (O) (P) (Q)
	straight	average	sloping

WITHERS

HEIGHT

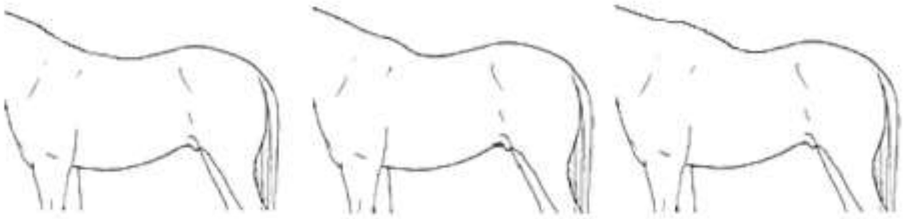

WHL	(A) (B) (C) (D) (E)	(F) (G) (H) (I) (L)	(M) (N) (O) (P) (Q)
	flat	average	high

CHEST

WIDTH

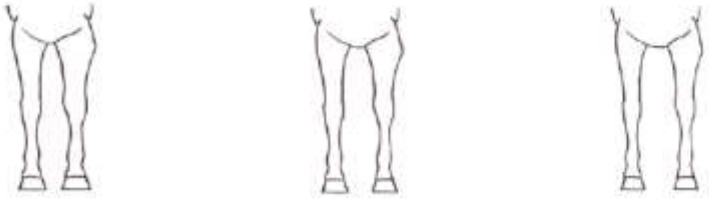

CWL	(A) (B) (C) (D) (E)	(F) (G) (H) (I) (L)	(M) (N) (O) (P) (Q)
	narrow	average	wide

By the Sport Horse Research Center – University of Perugia

CHEST

HEIGHT

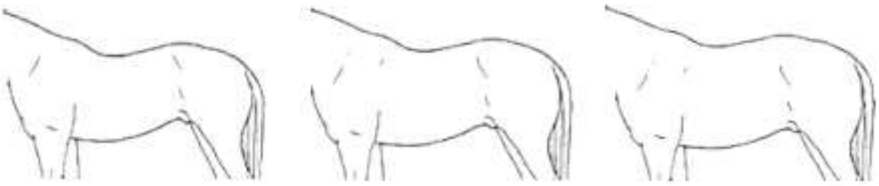

CHL

poorly depth	average	great depth
A B C D E	F G H I L	M N O P Q

LINE OF THE BACK

PROFILE

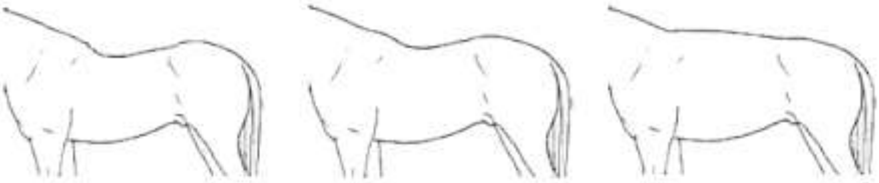

LBL

weak	average	roached
A B C D E	F G H I L	M N O P Q

LENGTH

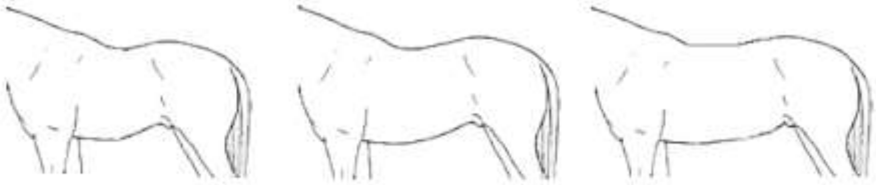

BLL

short	average	long
A B C D E	F G H I L	M N O P Q

LOIN

LENGTH

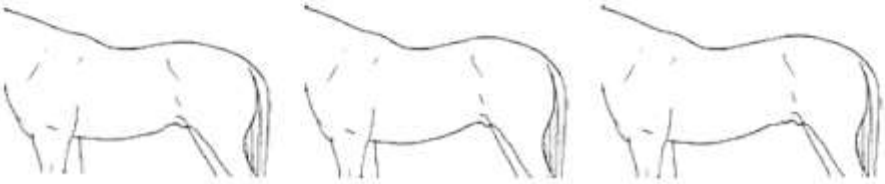

LLL

short	average	long
A B C D E	F G H I L	M N O P Q

SHAPE OF THE CROUP

By the Sport Horse Research Center – University of Perugia

POSITION

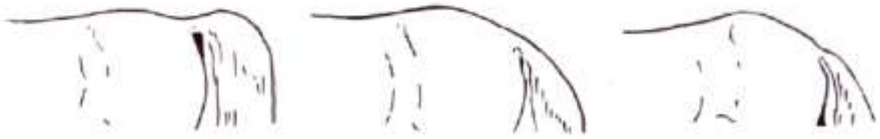

SCL

Ⓐ Ⓑ Ⓒ Ⓓ Ⓔ | Ⓕ Ⓖ Ⓗ Ⓘ Ⓛ | Ⓜ Ⓝ Ⓞ Ⓟ Ⓠ

straight average sloping

LENGTH

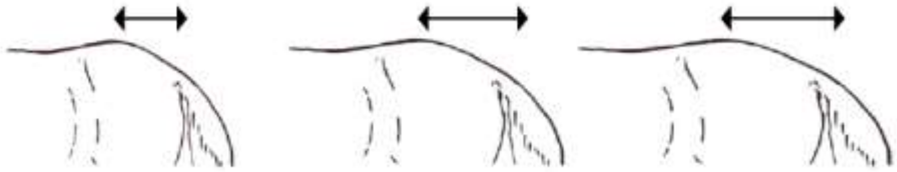

CLL

Ⓐ Ⓑ Ⓒ Ⓓ Ⓔ | Ⓕ Ⓖ Ⓗ Ⓘ Ⓛ | Ⓜ Ⓝ Ⓞ Ⓟ Ⓠ

short average long

WIDTH

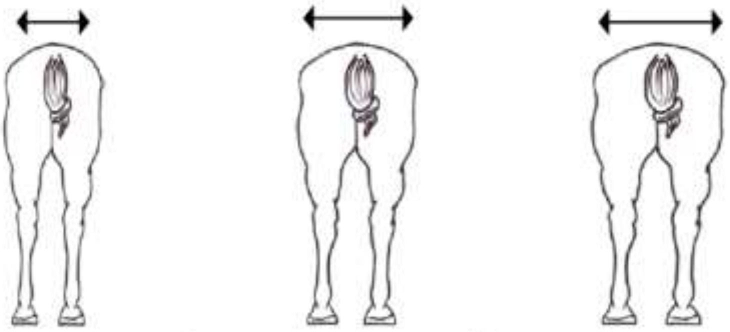

CrWL

Ⓐ Ⓑ Ⓒ Ⓓ Ⓔ | Ⓕ Ⓖ Ⓗ Ⓘ Ⓛ | Ⓜ Ⓝ Ⓞ Ⓟ Ⓠ

narrow average wide

PASTERN

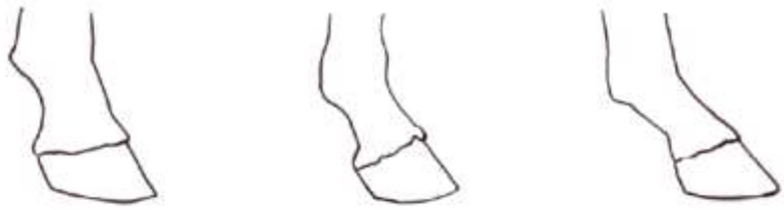

PAL

Ⓐ Ⓑ Ⓒ Ⓓ Ⓔ | Ⓕ Ⓖ Ⓗ Ⓘ Ⓛ | Ⓜ Ⓝ Ⓞ Ⓟ Ⓠ

short average long

LIMBS CONFORMATION

By the Sport Horse Research Center – University of Perugia

FORELEG FRONT VIEW

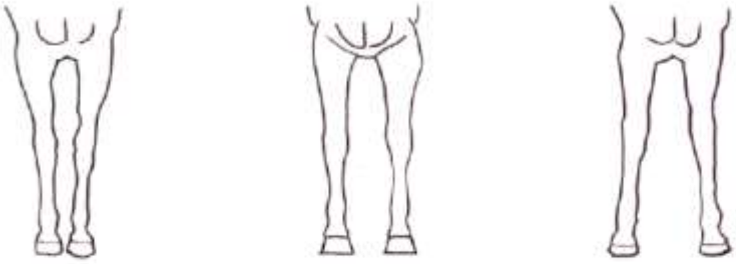

A B C D E | F G H I L | M N O P Q

FFBN

base narrow — average — wide

FORELEG FRONT VIEW

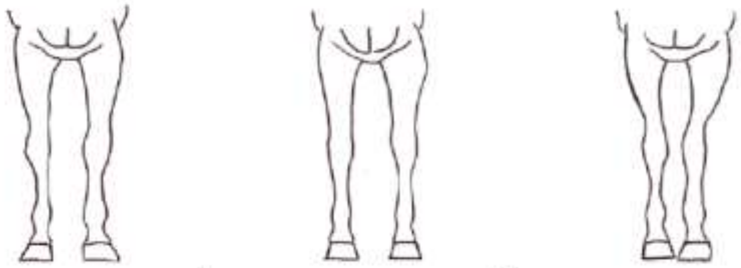

A B C D E | F G H I L | M N O P Q

FFTO

toes out — average — toes in

FORELEG FRONT VIEW

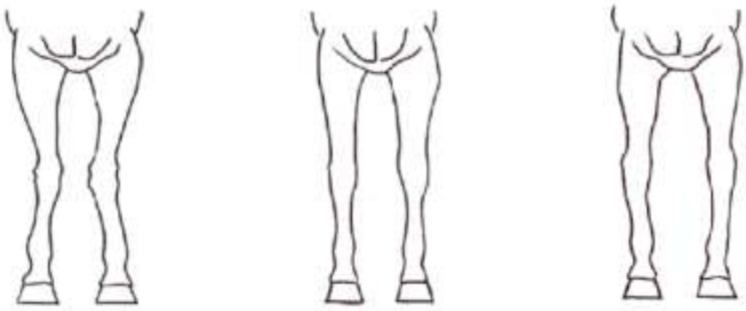

A B C D E | F G H I L | M N O P Q

FFKK

knock-knees — average — bowlegs

FORELEG SIDE VIEW

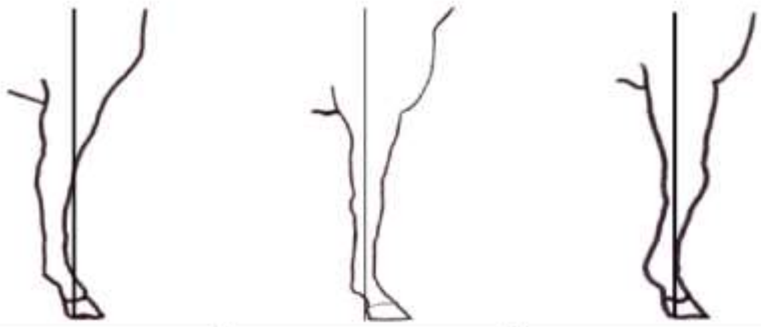

A B C D E | F G H I L | M N O P Q

FSCK

calf-kneed — average — buck-kneed

By the Sport Horse Research Center – University of Perugia

HINDLEG REAR VIEW

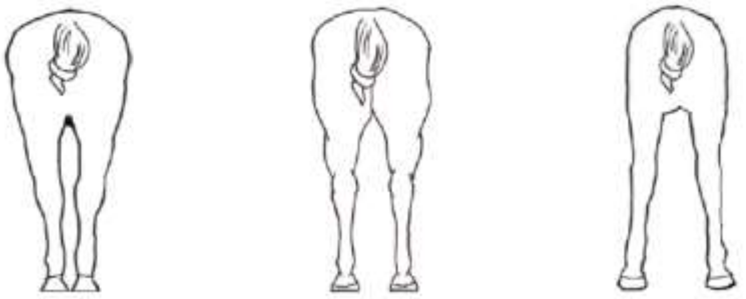

HLBN (A) (B) (C) (D) (E) | (F) (G) (H) (I) (L) | (M) (N) (O) (P) (Q)

base narrow | average | wide

HIND LEG REAR VIEW

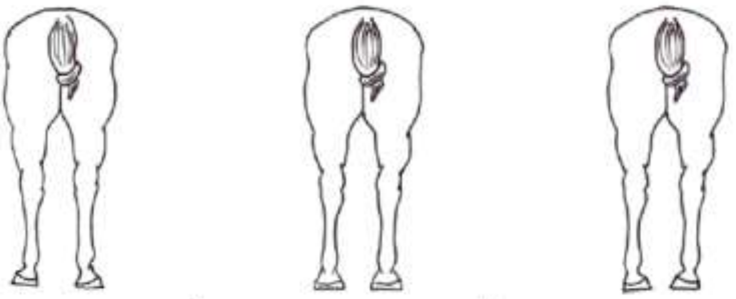

HLTO (A) (B) (C) (D) (E) | (F) (G) (H) (I) (L) | (M) (N) (O) (P) (Q)

toes out | average | toes in

HIND LEG REAR VIEW

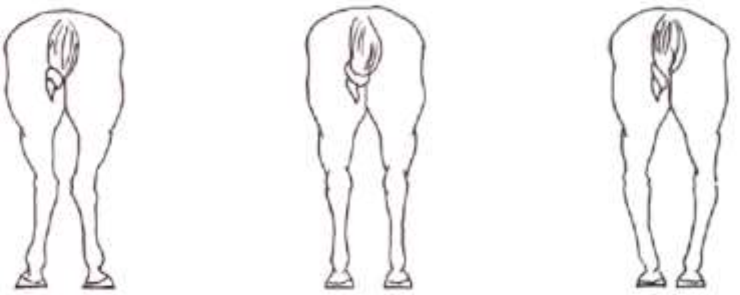

HLCH (A) (B) (C) (D) (E) | (F) (G) (H) (I) (L) | (M) (N) (O) (P) (Q)

cow hocked | average | bowlegged

HIND LEG SIDE VIEW

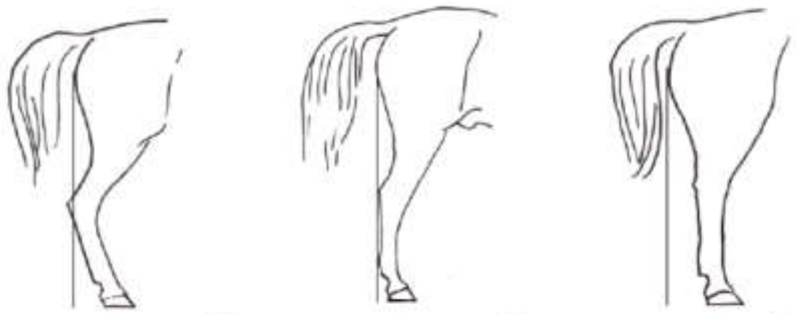

HLSH (A) (B) (C) (D) (E) | (F) (G) (H) (I) (L) | (M) (N) (O) (P) (Q)

sickle-hocked | average | too straight
