# Supplementary material for: Breeding Value Estimation Based on Morphological Evaluation of the Maremmano Horse Population through Factor Analysis
Source: Animals (Basel). 2024 Jul 31;14(15):2232. doi: 10.3390/ani14152232 (PMC11310958; doi:10.3390/ani14152232)
Supplement: Supplementary file 1 [file animals-14-02232-s001.zip › Additional_Table_1.pdf]

Distribution of the evaluated traits divided by age class

	HPL	HVL	NAL	SPL	WHL	LBL	SCL	NLL	CWL	SLL	CHL	BLL	LLL	CLL	CrWL	FFBN	FFTO	FFKK	FSCK	HLBN	HLTO	HLCH	HLSH	PAL	BCS
**Young**																									
mean	9	8	8	8	8	8	8	9	8	10	8	9	10	8	8	7	7	8	8	8	8	9	8	8	3
min	4	4	2	4	2	5	3	4	2	4	4	5	6	3	5	4	4	4	8	5	4	7	5	5	2
max	11	11	13	12	12	12	11	12	12	13	12	13	13	12	11	10	11	8	10	13	9	14	11	11	4
n = 38																									
**Mature**																									
mean	10	11	7	10	11	8	7	9	9	11	11	8	8	8	8	5	6	8	8	6	7	8	8	8	3
min	8	10	5	8	10	8	6	8	8	10	9	7	7	5	8	3	3	8	8	4	5	8	8	7	3
max	11	11	8	12	12	8	8	10	9	12	12	8	8	10	8	7	9	8	8	7	8	8	8	8	3
n = 2																									

Distribution of evaluated traits divided by body condition score (BCS)

	HPL	HVL	NAL	SPL	WHL	LBL	SCL	NLL	CWL	SLL	CHL	BLL	LLL	CLL	CrWL	FFBN	FFTO	FFKK	FSCK	HLBN	HLTO	HLCH	HLSH	PAL
**BCS = 2**																								
mean	11	8	2	5	2	10	5	4	2	10	4	13	13	5	5	8	5	8	8	8	4	14	5	8
min	11	8	2	5	2	10	5	4	2	10	4	13	13	5	5	8	5	8	8	8	4	14	5	8
max	11	8	2	5	2	10	5	4	2	10	4	13	13	5	5	8	5	8	8	8	4	14	5	8
n = 1																								
**BCS = 3**																								
mean	9	8	8	8	9	8	9	8	8	10	9	9	9	8	8	7	7	8	8	7	7	8	8	8
min	4	4	3	4	5	5	3	4	5	4	5	5	6	3	5	3	3	4	8	4	5	7	6	5
max	11	11	13	12	12	8	11	11	11	13	12	12	12	12	11	10	11	8	10	10	9	12	11	11
n = 33																								
**BCS = 4**																								
mean	8	8	11	6	8	8	6	11	6	8	10	6	10	8	8	8	10	8	8	6	8	11	10	9
min	8	8	11	6	8	8	6	11	6	8	10	6	10	8	8	8	10	8	8	6	8	11	10	9
max	8	8	11	6	8	8	6	11	6	8	10	6	10	8	8	8	10	8	8	6	8	11	10	9
n = 1																								

Distribution of the evaluated traits divided by judge

	HPL	HVL	NAL	SPL	WHL	LBL	SCL	NLL	CWL	SLL	CHL	BLL	LLL	CLL	CrWL	FFBN	FFTO	FFKK	FSCK	HLBN	HLTO	HLCH	HLSH	PAL	BCS
**Judge (A)**																									
mean	8	8	8	8	10	8	10	9	7	10	9	8	10	7	8	8	8	8	8	7	7	9	9	9	3
min	7	7	5	5	8	7	6	8	6	6	8	6	10	6	6	7	5	8	8	5	6	8	8	8	3
max	11	9	12	11	12	8	11	11	8	13	10	10	10	9	8	8	10	8	8	8	9	11	10	9	4
n = 6																									
**Judge (B)**																									
mean	9	8	8	9	8	8	9	9	8	10	9	9	9	8	8	7	7	8	8	8	8	8	8	8	3
min	8	5	6	7	5	5	6	6	5	7	6	8	8	5	5	4	4	4	8	6	6	7	7	7	3
max	11	11	9	12	11	8	11	11	11	13	12	11	11	8	11	9	11	8	8	10	8	12	8	11	3
n = 13																									
**Judge (C)**																									
mean	11	11	5	12	12	8	8	8	8	12	12	8	8	5	8	3	3	8	8	4	5	8	8	8	3
min	11	11	5	12	12	8	8	8	8	12	12	8	8	5	8	3	3	8	8	4	5	8	8	8	3
max	11	11	5	12	12	8	8	8	8	12	12	8	8	5	8	3	3	8	8	4	5	8	8	8	3
n = 1																									
**Judge (D)**																									
mean	9	8	7	9	7	9	8	11	8	10	6	9	11	7	8	7	7	8	8	9	8	8	8	8	0
min	8	5	5	5	4	8	5	10	4	8	4	8	10	5	5	4	5	8	8	8	8	8	8	8	0
max	10	11	8	11	10	12	11	12	12	11	8	11	11	9	11	8	8	8	8	13	8	8	8	8	0
n = 5																									
**Judge (E)**																									
mean	9	7	8	7	8	8	8	7	8	10	8	10	10	9	8	8	7	8	8	8	7	9	8	8	3
min	4	4	2	4	2	6	3	4	2	4	4	5	6	3	5	6	5	8	8	5	4	8	5	5	2
max	11	10	13	11	11	10	11	11	11	12	11	13	13	12	11	10	11	8	10	10	8	14	11	10	3
n = 14																									
**Judge (F)**																									
mean	8	10	8	8	10	8	6	10	9	10	9	7	7	10	8	7	9	8	8	7	8	8	8	7	3
min	8	10	8	8	10	8	6	10	9	10	9	7	7	10	8	7	9	8	8	7	8	8	8	7	3
max	8	10	8	8	10	8	6	10	9	10	9	7	7	10	8	7	9	8	8	7	8	8	8	7	3
n = 1																									
