# Supplementary figures and images for: Breeding Value Estimation Based on Morphological Evaluation of the Maremmano Horse Population through Factor Analysis
Source: Animals (Basel). 2024 Jul 31;14(15):2232. doi: 10.3390/ani14152232 (PMC11310958; doi:10.3390/ani14152232)

**Figure S2:** Correlation coefficients between the judges' scoring for each variable (n=600).

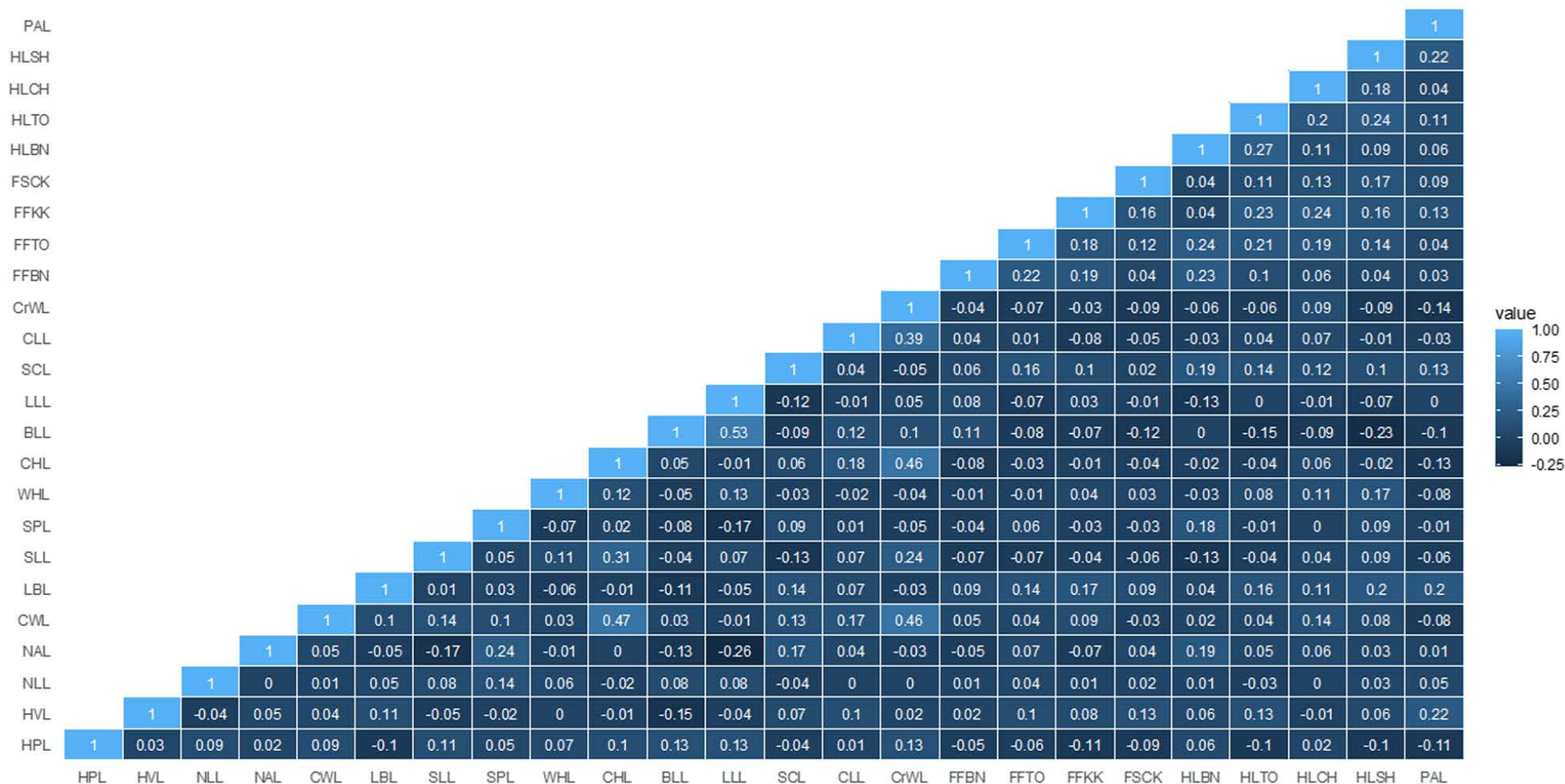

Supplement: Supplementary file 1 [file animals-14-02232-s001.zip › Additional_Figure_2.pdf]
